# Translation Inhibitors Induce Formation of Cholesterol Ester-Rich Lipid Droplets

**DOI:** 10.1371/journal.pone.0042379

**Published:** 2012-08-03

**Authors:** Michitaka Suzuki, Yuki Ohsaki, Tsuyako Tatematsu, Yuki Shinohara, Takashi Maeda, Jinglei Cheng, Toyoshi Fujimoto

**Affiliations:** Department of Anatomy and Molecular Cell Biology, Nagoya University Graduate School of Medicine, Nagoya, Japan; Nihon University School of Medicine, Japan

## Abstract

Lipid droplets (LDs) in non-adipocytes contain triglycerides (TG) and cholesterol esters (CE) in variable ratios. TG-rich LDs are generated when unsaturated fatty acids are administered, but the conditions that induce CE-rich LD formation are less well characterized. In the present study, we found that protein translation inhibitors such as cycloheximide (CHX) induced generation of CE-rich LDs and that TIP47 (perilipin 3) was recruited to the LDs, although the expression of this protein was reduced drastically. Electron microscopy revealed that LDs formed in CHX-treated cells possess a distinct electron-dense rim that is not found in TG-rich LDs, whose formation is induced by oleic acid. CHX treatment caused upregulation of mTORC1, but the CHX-induced increase in CE-rich LDs occurred even when rapamycin or Torin1 was given along with CHX. Moreover, the increase in CE was seen in both wild-type and autophagy-deficient Atg5-null mouse embryonic fibroblasts, indicating that mTORC1 activation and suppression of autophagy are not necessary to induce the observed phenomenon. The results showed that translation inhibitors cause a significant change in the lipid ester composition of LDs by a mechanism independent of mTORC1 signaling and autophagy.

## Introduction

The lipid droplet (LD) is a subcellular structure that exists in a range of organisms from archaea to mammals. The LD used to be regarded as an inert lipid depot, but recent studies have revealed that it is an active organelle engaged in a wide range of activities [1], [2], [3]. The main function of LDs is to store lipids and to supply them for various cellular needs, such as β-oxidation, membrane biogenesis, and lipoprotein synthesis.

The structure of the LD consists of a core of lipid esters and a surface lined with a phospholipid monolayer [4], [5]. In white adipocytes, the lipid ester core consists almost exclusively of triglycerides (TG), whereas in many non-adipocytes LDs contain both TG and cholesterol esters (CE) in various ratios [6]. TG synthesis is facilitated in the presence of excess fatty acids. In many non-adipocytes in culture, only a small number of LDs exist under normal conditions, but the addition of unsaturated fatty acids such as oleic acid to the medium induces abundant TG-rich LDs. CE metabolism has been studied most actively using macrophage foam cells, which take up significant quantities of plasma lipoproteins [7]; in contrast, the general conditions that induce CE accumulation in other cell types are not well known.

Degradation mechanisms have also been more thoroughly analyzed for TG than for CE. The regulatory mechanism of cytosolic lipases, including adipocyte triglyceride lipase (ATGL) and hormone-sensitive lipase (HSL), has been rapidly unveiled [8]. In contrast, the enzymes engaged in CE hydrolysis have not been firmly established, even in macrophage foam cells [9]. A recent study revealed that autophagy is involved in the degradation of LDs in hepatocytes [10], but it is not yet known in detail whether and to what extent this process is active in other cell types.

In the present study, we found that treatment with protein translation inhibitors causes a significant increase in CE-rich LDs. Translation inhibitors are frequently used in cell biological experiments, but the effect observed in the present study has not been given attention in the past. Earlier studies showed that treatment with cycloheximide suppresses autophagy [11], [12]. More recently, inhibition of protein synthesis was shown to activate mTORC1 [13], [14], [15]. We aimed to investigate whether the increase in CE-rich LDs that results from treatment with translation inhibitors was caused by mTORC1 activation and/or suppression of autophagy.

**Figure 1:**
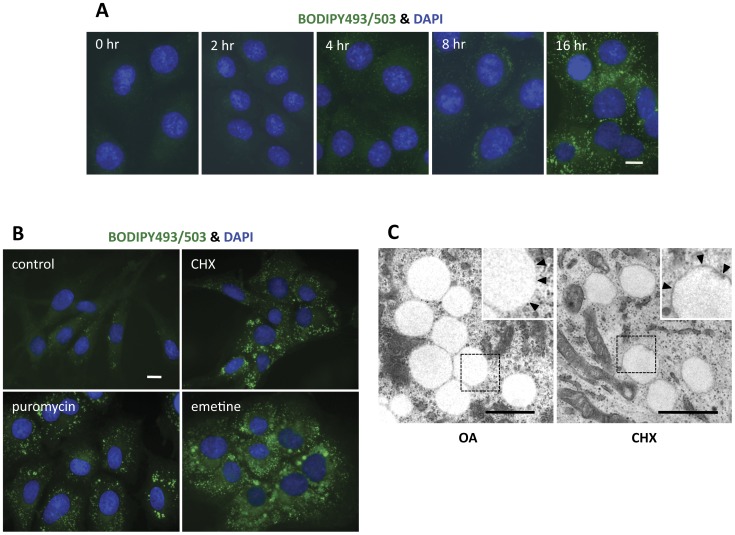
Translation inhibitors induced an increase in cytoplasmic lipid droplets. (A) Time course of the LD increase in 3Y1 cells treated with 10 µg/ml cycloheximide (CHX). LDs and nuclei were stained with BODIPY493/503 and DAPI, respectively. Small LDs were detectable as early as 4 hr after the addition of CHX, but the increase in LDs became more prominent with longer incubation. Bar, 10 µm. (B) Effect of different translation inhibitors. 3Y1 cells under normal culture conditions were compared with those treated with 10 µg/ml CHX, 2 µM puromycin, or 20 µM emetine for 18 hr. LDs increased in both size and number upon treatment with any of the reagents. Bar, 10 µm. (C) Electron microscopy. LDs in 3Y1 cells that were treated with either 10 µg/ml CHX or 0.4 mM oleic acid (OA) for 18 hr were observed as round electron-lucent structures. High-magnification figures of the rectangular areas are shown in the inset. Notably, the perimeters of LDs induced by CHX were lined with an electron-dense rim, which was not seen in those induced by OA (arrowheads in the inset). Bar, 1 µm.

## Materials and Methods

### Cells

Mouse embryonic fibroblasts (MEF) that were obtained from *atg5^+/+^* and *atg5^−/−^* mice and immortalized using an SV40 T-antigen [16] were kindly provided by Dr. Noboru Mizushima (Tokyo Medical and Dental University). 3Y1, 293A, and Huh7 cells were obtained from the Japanese Collection of Research Bioresources Cell Bank. The cells were cultured in Dulbecco’s minimum essential medium supplemented with 10% fetal calf serum and antibiotics at 37°C in a humidified atmosphere containing 5% CO_2_. In some experiments, cells were treated with 0.4 mM oleic acids (OA) (Sigma-Aldrich, St. Louis, MO, USA) complexed with fatty acid-free bovine serum albumin (Wako Chemicals, Ltd., Osaka, Japan) at a molar ratio of 6∶1 [17] to increase the TG content. In others, cholesterol complexed with methyl-β-cyclodextrin (MβCD) [18] at the final concentration of approximately 50 µg cholesterol/ml was added to the culture medium to increase the cellular CE content.

### Antibodies and Reagents

Mouse anti-ADRP (Progen Pharmaceuticals, Toowong, Australia), rabbit anti-p70 ribosomal S6 kinase (S6K) and mouse anti-phospho-S6K (Thr389) (Cell Signaling Technology, Danvers, MA, USA), mouse anti-lysosomal-associated membrane protein 1 (Lamp1; clone H4A3) (Developmental Studies Hybridoma Bank, Iowa City, IA, USA), rabbit anti-LC3 (MBL, Woburn, MA, USA), and rabbit anti-β-actin (Sigma-Aldrich) were purchased from the indicated suppliers. Secondary antibodies conjugated with fluorochromes were obtained from Jackson ImmunoResearch Laboratories (West Grove, PA, USA) and Invitrogen (Carlsbad, CA, USA). Torin1 was kindly provided by Dr. David Sabatini (Whitehead Institute for Biomedical Research). Other reagents were purchased from Sigma-Aldrich.

**Figure 2:**
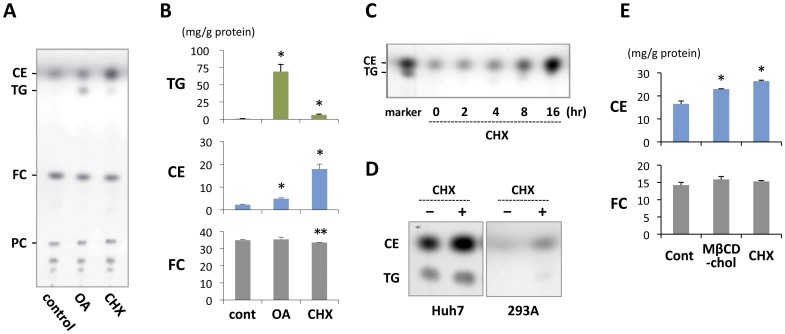
CHX treatment caused an increase in cellular cholesterol ester content. (A) Thin layer chromatography (TLC) of the total lipid extract from 3Y1 cells. Cholesterol esters (CE) increased significantly after treatment with 10 µg/ml CHX for 18 hr, whereas triglycerides (TG) did not show a significant change. In contrast, in cells treated with 0.4 mM OA, TG rather than CE increased. Lipids extracted from cells with an equal protein content were compared. (B) Quantification of TG, CE, and free cholesterol (FC) in 3Y1 cells. The experimental conditions were the same as in (A). CHX increased CE significantly, whereas OA induced a prominent increase in TG. The FC content was equivalent in the three samples. Mean ± standard deviation (SD) is shown. The difference from the control sample was examined by Student’s *t* test (*p<0.01, **p<0.05). (C) Time course of the CE increase after CHX treatment. The increase in CE in 3Y1 cells was detectable by means of TLC as early as 4 hr after treatment with 10 µg/ml CHX. (D) TLC of the total lipid extract from Huh7 and 293A cells treated without or with 10 µg/ml CHX for 18 hr. The increase in CE was observed in both cell types. (E) Quantification of CE and FC in Huh7 cells that were treated without or with 0.25 mM methyl-β-cyclodextrin-cholesterol complex (MβCD-FC) or with 10 µg/ml CHX for 18 hr. Both treatments increased CE without affecting the FC content. Mean ± SD is shown. The difference from the control sample was examined by Student’s *t* test (*p<0.01).

### Fluorescence Microscopy

Cells were fixed with 3% formaldehyde in 0.1 M phosphate buffer for more than 30 min. LDs and nuclei were stained with BODIPY493/503 (Invitrogen) and 4,6-diamidino-2-phenylindole (DAPI, Sigma-Aldrich), respectively. All treatments were performed at room temperature. Images were captured by an Apotome/Axiovert 200M microscope (Carl Zeiss, Oberkochen, Germany) using an Apochromat 63x objective lens with a 1.40 numerical aperture. For quantification of labeling intensity, more than 12 randomly selected areas in each sample were photographed under the same microscopic settings. The areas in which the labeling intensity was above a certain threshold per unit cell area were measured with ImageJ software (http://rsb.info.nih.gov/ij/). The color, brightness, and contrast of the images were adjusted using Adobe Photoshop CS3 for presentation.

### Western Blotting

Cells were lysed in sample buffer containing 2.5% SDS and the protein concentration was analyzed by BCA assay. Samples (10–30 µg) were electrophoresed by SDS-PAGE and Western blot signals were detected by chemiluminescence.

### Thin-layer Chromatography (TLC)

The total lipids of cells were extracted with hexane and isopropanol [19]. The lipids obtained from samples containing equivalent amount of proteins were developed on an HPTLC plate (Silica Gel 60, Merck, Darmstadt, Germany) with chloroform-methanol-acetic acid-formic acid-water (35:15:6:2:1) and then with hexanes-diisopropyl ether-acetic acid (65:35:2). Each plate was charred with cupric acetate-phosphoric acid [20].

### Quantification of Lipids

Lipids were extracted as described above and free cholesterol (FC) and TG were measured by colorimetric enzyme assays using reagents for clinical tests for serum lipids (Determiner L FC, Determiner L TG II, Kyowa Medex Co., Ltd., Tokyo, Japan) as described elsewhere [21]. In brief, samples and standard lipid solutions were incubated with the reagents in a 96-well plate and the absorbance was measured with a fluorospectrophotometer at two different wavelengths. To measure total cholesterol, i.e., FC plus CE, the sample was pretreated with cholesterol esterase before quantification using the Determiner L FC test kit. In each experiment, lipids extracted from duplicate cell samples were measured and all experiments were repeated more than two times. Representative results are presented in the figures.

**Figure 3:**
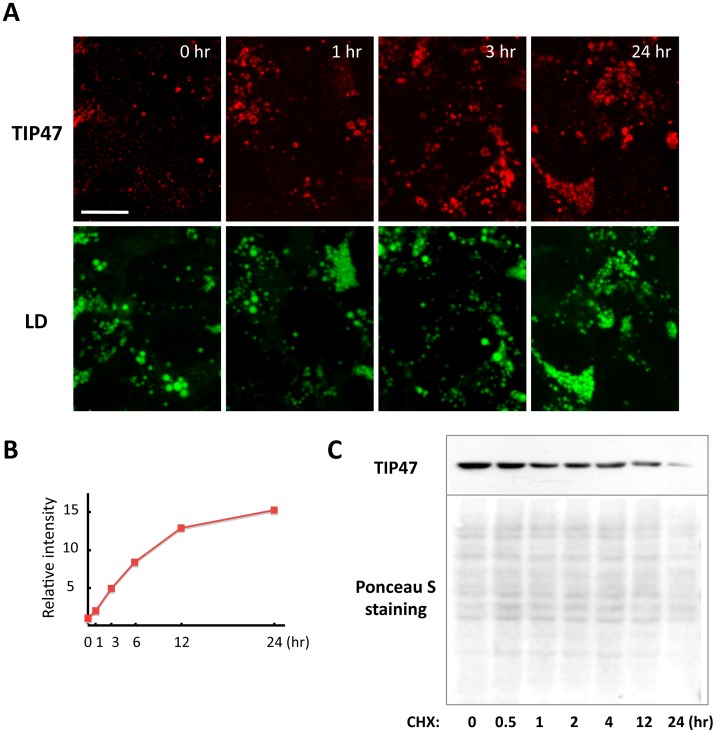
TIP47 was recruited to LDs by CHX treatment. (A) Huh7 cells were treated with 10 µg/ml CHX for up to 24 hr and doubly labeled with anti-TIP47 antibody (red) and BODIPY493/503 (green). Many LDs existed in Huh7 cells even under normal culture conditions. CHX treatment induced a significant recruitment of TIP47 to LDs, whereas the increase of LD was moderate. Bar, 10 µm. (B) The relative intensity of TIP47 immunofluorescence labeling was quantified in more than 12 random fields in each sample. The labeling intensity increased significantly by the CHX treatment. (C) Western blotting of TIP47. The TIP47 protein in Huh7 cells was decreased by the CHX treatment. The Ponceau S staining confirmed that equivalent amounts of proteins were blotted to the nitrocellulose membrane for each sample. Each lane was loaded with 10 µg protein.

### Electron Microscopy

Cells cultured on coverslips were fixed with 2.5% glutaraldehyde in 0.1 M sodium cacodylate buffer and post-fixed in a mixture of 1% osmium tetroxide and 0.1% potassium ferrocyanide in the same buffer [22]. After dehydration through an ethanol series, samples were embedded in Quetol 812 resin. Ultrathin sections were observed using a JEOL 1400CX electron microscope (Tokyo, Japan) at 100 kV.

## Results

### Translation Inhibitors Induce LD Formation

Rat 3Y1 fibroblasts were used in most experiments. 3Y1 cells have few small LDs when cultured in normal culture medium. When treated with the translation inhibitor cycloheximide (CHX) for several hours, we found that 3Y1 cells tend to contain many LDs. Cells stained with BODIPY 493/503 showed a detectable increase in LDs at 4 hr by fluorescence microscopy; this increase became more prominent after 16 hr of CHX treatment (Fig. 1A). Puromycin and emetine, which inhibit protein translation by different mechanisms [23], also induced a similar increase in LDs in 3Y1 cells (Fig. 1B). The increase in LDs induced by CHX was not limited to 3Y1 cells, but was observed in other cell types as well (data not shown).

Under electron microscopic examination, LDs generated in the presence of CHX were observed as electron-lucent round structures (Fig. 1C) and appeared similar to those that had formed in the presence of excess OA. Observation under a higher magnification, however, revealed that only CHX-induced LDs had a thin electron-dense line in their rims (inset of Fig. 1C). As shown below, LDs in CHX-treated cells were enriched with CE and the rim may be related to the propensity of sterol esters to make concentric layers [24]. CE-rich LDs without such a rim were, however, also observed [22], [25], indicating that it is not a general characteristic of theirs.

**Figure 4:**
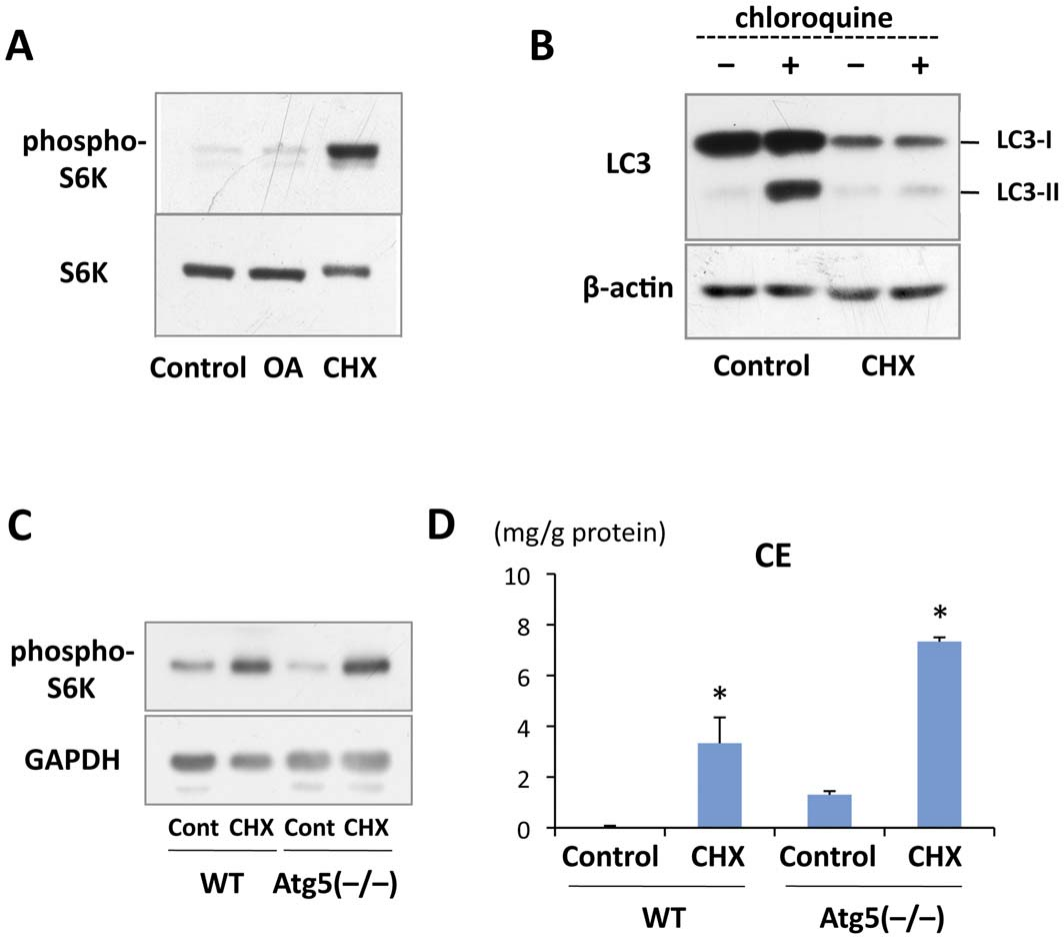
CHX caused the CE increase even in autophagy-deficient cells. (A) 3Y1 cells were treated without or with either 10 µg/ml CHX or 0.4 mM OA for 18 hr. CHX caused a significant increase in phospho-S6K. (B) The autophagic flux was examined by incubating 3Y1 cells with 20 µM chloroquine for 1 hr immediately before sample preparation. Chloroquine caused a significant increase in LC3-II in the control, but not in cells pretreated with 10 µg/ml CHX for 18 hr. (C) Wild-type and Atg5-null MEF were treated without or with 10 µg/ml CHX for 18 hr. The increase in phospho-S6K was observed in a comparable degree in both cell lines. GAPDH was probed as a loading control. (D) Wild-type and Atg5-null MEF were treated without or with 10 µg/ml CHX for 18 hr. CE increased significantly in response to CHX treatment in both cell lines. Mean ± SD is shown. The difference from the respective control was examined by Student’s *t* test (*p<0.01).

### CE Increased by Inhibition of Translation

In most non-adipocytes, LDs contain TG and CE in various proportions [6]. To examine which of the lipid esters are responsible for the LD increase induced by CHX, lipids extracted from cells were examined. Both TLC and colorimetric enzyme assays showed that, after CHX treatment, CE increased to a much higher degree than TG did (Figs. 2A, 2B). In cells treated with OA, in contrast, the increase in TG was predominant. The time course of the CE increase was similar to that of the LD increase, and the CE increase was detectable at 4 hr by means of TLC (Fig. 2C).

A similar increase in CE was observed in other cell types treated with CHX (Fig. 2D). Huh7 cells are different from 3Y1 in that Huh7 cells harbor many large LDs even under normal culture conditions and contain both TG and CE abundantly. Yet in Huh7 cells also, CHX treatment induced a significant increase in CE with a much smaller effect on TG (Figs. 2D, 2E). The increase of CE caused by the CHX treatment became larger than that in cells loaded with excessive FC with the addition of 0.25 mM MβCD-cholesterol complex to the culture medium (Fig. 2E).

**Figure 5:**
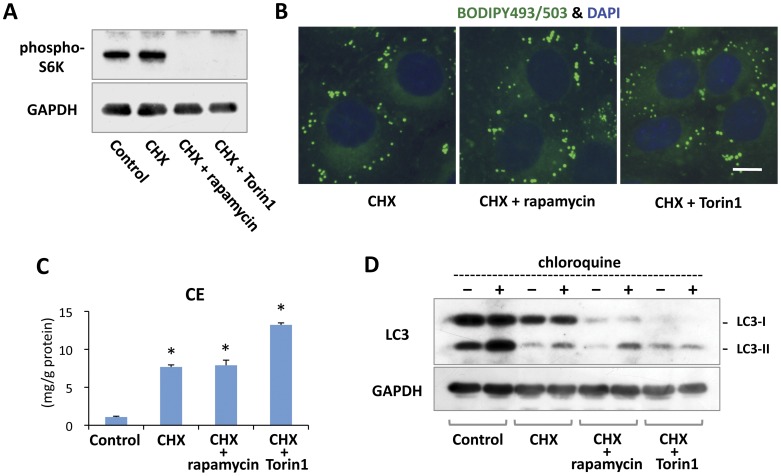
CHX induced increases in LDs and CE even in the presence of mTORC1 inhibitors. (A) 3Y1 cells were treated without or with 10 µg/ml CHX, 10 µg/ml CHX and 0.4 µM rapamycin, or 10 µg/ml CHX and 0.25 µM Torin1 for 8 hr. Addition of rapamycin or Torin1 decreased phospho-S6K significantly. GAPDH is shown as a loading control. Each lane was loaded with 20 µg protein. (B) 3Y1 cells were treated with 10 µg/ml CHX alone or together with 0.4 µM rapamycin or 0.25 µM Torin1 for 18 hr. LDs increased to a similar degree irrespective of the presence of rapamycin or Torin1. Bar, 10 µm. (C) 3Y1 cells were treated without or with 10 µg/ml CHX, 10 µg/ml CHX and 0.4 µM rapamycin, or 10 µg/ml CHX and 0.25 µM Torin1 for 18 hr. CE increased in response to CHX treatment even when rapamycin or Torin1 was given simultaneously. Mean ± SD is shown. The difference from the control sample was examined by Student’s *t* test (*p<0.01). (D) 3Y1 cells were treated in the same manner as in Fig. 5A. The autophagic flux was examined by adding 20 µM chloroquine for 1 hr immediately before sample preparation. A low level of LC3-II increase was caused by chloroquine in samples treated with CHX alone or CHX and rapamycin, but not in samples treated with CHX and Torin1. GAPDH is shown as a loading control. Each lane was loaded with 50 µg protein.

### LD-associated TIP47 Increased Despite a Decrease in Total TIP47

TIP47 (perilipin 3) is a perilipin-family protein. Huh7 cells under normal culture conditions have many LDs and express abundant TIP47, but little TIP47 was observed in LDs by immunofluorescence microscopy [26]. This is in contrast to ADRP (perilipin 2), which shows intense labeling around LDs [26]. When cells were incubated with CHX, labeling of TIP47 in LDs started to increase as early as 1 hr and became more intense at 12–24 hr (Fig. 3A). The increase in LDs was moderate after CHX treatment, whereas the intensity of TIP47 labeling increased drastically (Fig. 3B). ADRP labeling intensity did not change significantly due to CHX treatment (data not shown). The increase in TIP47 labeling after CHX treatment was peculiar if we consider that TIP47 per unit protein weight decreased drastically during the same time (Fig. 3C). The amount of TIP47 per cell was thought to decrease as well because the total protein per cell remained almost the same even after CHX treatment (data not shown).

### CHX Increased CE-rich LDs Even in Autophagy-deficient Cells

With regard to the mechanism underlying the LD increase in CHX-treated cells, we thought that mTORC1 activation [13], [14], [15] and/or suppression of autophagy [11], [12] may be involved. In fact, an increase in phosphorylated S6K, an mTORC1 substrate, was confirmed to occur in 3Y1 cells treated with CHX (Fig. 4A). An important consequence of mTORC activation is inhibition of autophagy (for a recent review, see Jung et al., 2010), and the autophagic flux in 3Y1 cells was in fact suppressed significantly by CHX (Fig. 4B).

To examine the possibility that down-regulation of autophagy by CHX caused the increase in CE-rich LDs, we turned to autophagy-deficient MEF taken from Atg5 knockout mice [16]. It was confirmed that mTORC1 was activated similarly by CHX in wild-type and Atg5-null MEF (Fig. 4C). However, CE and LDs were observed to increase as a result of CHX treatment in both cell types (Fig. 4D; data not shown). These results demonstrated that the CHX-induced increase in CE-rich LDs does not depend on suppression of autophagy. It is notable, however, that significantly larger amounts of CE were found in Atg5-null cells than in wild-type cells, both without and with CHX treatment (Fig. 4D), indicating that autophagy may also be engaged in degrading CE-rich LDs.

### CE-rich LDs Increased Even When mTORC1 was Inhibited

Since mTORC1 is a hub of many intracellular signaling pathways, its activation could cause various other effects in addition to autophagic suppression [27]. To test whether the CHX-induced LD increase occurred as a result of mTORC1 activation, cells were treated with either rapamycin or Torin1 along with CHX. It was confirmed that phosphorylation of S6K was suppressed effectively by either 0.4 µM rapamycin or 0.25 µM Torin1 (Fig. 5A). Even in the presence of these mTORC1 inhibitors, CHX induced a significant increase in LDs (Fig. 5B) and CE (Fig. 5C), indicating that mTORC1 activation is not involved in the mechanism.

Interestingly, in cells treated with rapamycin or Torin1 along with CHX, even though mTORC1 was inhibited, autophagy was not activated (Fig. 5D): the autophagic flux was low (rapamycin) or appeared nonexistent (Torin1). The seemingly complete suppression of autophagy with CHX and Torin1 may explain the significantly higher CE increase in those cells than that in cells treated with CHX alone or with CHX and rapamycin (Fig. 5C). On the other hand, in the absence of CHX, Torin1 alone induced a slight but significant increase of CE (Fig. S1). The result suggested that Torin1 enhanced the CHX-induced CE increase by suppressing mTORC2, which is likely to be a negative regulator of lipid accumulation [28], [29].

## Discussion

In the present study, we found that protein translation inhibitors cause a significant increase in CE-rich LDs. Because translation inhibitors are known to cause mTORC1 activation [13], [14], [15] and autophagy suppression [11], [12], we initially supposed that those processes were responsible for the increase in CE-rich LDs. Yet this increase in CE and LDs was observed even in the presence of mTORC1 inhibitors and in autophagy-deficient cells, indicating the engagement of other mechanisms. As a possible cause of the observed phenomena, we speculate that translation inhibitors may cause a down-regulation of CE hydrolysis: that is, CE hydrolytic enzymes may have a relatively short half-life and may decrease quickly when protein synthesis is suppressed. Hormone-sensitive lipase (HSL) may be engaged in CE hydrolysis [30], but if its decrease were the main cause of the CE increase in CHX-treated cells, TG would be expected to increase simultaneously, and this was not observed in the present experiment. Other than HSL, several neutral CE hydrolases have been reported to be critical for CE digestion in macrophage foam cells, but their role in other cell types is not clear [9]. Thus we are yet to examine the aforementioned possibility.

We observed that the CHX-induced increase in CE and LDs also occurs in autophagy-deficient Atg5-null MEF, but this does not preclude the possibility that autophagy is involved in CE metabolism and LD turnover [10]. In fact we found that significantly larger amounts of CE were observed in Atg5-deficient MEF than in wild-type MEF both before and after CHX treatment (Fig. 4D). Moreover, the seemingly complete suppression of the autophagic flow in cells treated with CHX and Torin1 caused a significantly higher increase of CE than in cells treated with CHX alone, in which a low level of autophagy was occurring (Fig. 5C, 5D). These results showed that autophagic suppression is not the main cause of the CE increase induced by CHX, but nonetheless they also indicated that autophagy is an important mechanism of CE degradation.

LDs that consist predominantly of TG in white adipocytes are degraded effectively by the sequential action of ATGL, HSL, and monoacylglycerol lipase [8]. To degrade LDs that are enriched with CE, however, lysosomal acid lipase, which has CE hydrolytic activity, may be involved, as it is for degradation of cholesterol-loaded macrophages [31]. For LDs containing CE and TG in comparable amounts, CE hydrolysis may be a prerequisite for effective TG degradation because CE may surround the TG core, forming concentric layers on the surface [24]. In this context, it is notable that the deficiency of lysosomal acid lipase that characterizes Wolman disease manifests as an accumulation of CE as well as TG [32].

It was surprising that, upon treatment with translation inhibitors, TIP47 was recruited to the CE-rich LDs even though the total amount of TIP47 decreased drastically. TIP47 was previously shown to be recruited to TG-rich LDs induced by unsaturated fatty acids, but in such cases the overall expression of TIP47 also increased [26]. The present result indicates that TIP47 recruitment to LDs does not depend on the increased expression of TIP47 or on the composition of the lipid esters in LDs; rather, it is directly related to the increment of lipid esters. On the other hand, the increased recruitment of TIP47 to LDs should reduce TIP47 in the soluble cytoplasmic fraction, especially when the total amount is downregulated. Although the non-LD function of TIP47 remains controversial [33], [34], it must be determined whether any result seen in the presence of translation inhibitors can be explained by a decrease in TIP47 in the cytoplasm.

The phenomena observed in the present study need to be taken into account in interpreting experimental results obtained using translation inhibitors. Yet the implications of this study are not limited to such artificial conditions, given that, in cells exposed to various stresses, protein synthesis is suppressed [35] and LDs increase [36], [37]. LDs that increase in cultured cells under ER stress are enriched with CE (Ohsaki et al., unpublished observation). The detailed mechanism underlying CE-rich LD formation as well as the impact of this process are worthy of further studies in this context.

## Supporting Information

Figure S1 **Quantification of CE.** 3Y1 cells were treated with or without 0.4 µM rapamycin or 0.25 µM Torin1 for 18 hr. CE showed an increase caused by either rapamycin or Torin1, but only the increase caused by Torin1 was significant (*p<0.05; Student’s *t* test). Mean ± SD is shown.
(TIF)

## References

[1] Walther TC, Farese RV Jr (2009) The life of lipid droplets. Biochim Biophys Acta 1791: 459–466. 19041421 10.1016/j.bbalip.2008.10.009 PMC2782899

[2] Fujimoto T, Parton RG (2011) Not just fat: the structure and function of the lipid droplet. Cold Spring Harb Perspect Biol 3. 10.1101/cshperspect.a004838 PMC3039932 21421923

[3] Murphy DJ (2011) The dynamic roles of intracellular lipid droplets: from archaea to mammals. Protoplasma. 10.1007/s00709-011-0329-7 22002710

[4] Murphy DJ, Vance J (1999) Mechanisms of lipid-body formation. Trends Biochem Sci 24: 109–115. 10203758 10.1016/s0968-0004(98)01349-8

[5] Tauchi-Sato K, Ozeki S, Houjou T, Taguchi R, Fujimoto T (2002) The surface of lipid droplets is a phospholipid monolayer with a unique Fatty Acid composition. J Biol Chem 277: 44507–44512. 12221100 10.1074/jbc.M207712200

[6] Fujimoto T, Ohsaki Y, Cheng J, Suzuki M, Shinohara Y (2008) Lipid droplets: a classic organelle with new outfits. Histochem Cell Biol 130: 263–279. 18546013 10.1007/s00418-008-0449-0 PMC2491702

[7] Li AC, Glass CK (2002) The macrophage foam cell as a target for therapeutic intervention. Nat Med 8: 1235–1242. 12411950 10.1038/nm1102-1235

[8] Zechner R, Madeo F (2009) Cell biology: Another way to get rid of fat. Nature 458: 1118–1119. 19407787 10.1038/4581118a

[9] Suzuki M, Shinohara Y, Ohsaki Y, Fujimoto T (2011) Lipid droplets: size matters. J Electron Microsc (Tokyo) 60 Suppl 1 S101–116. 21844583 10.1093/jmicro/dfr016

[10] Singh R, Kaushik S, Wang Y, Xiang Y, Novak I, et al (2009) Autophagy regulates lipid metabolism. Nature 458: 1131–1135. 19339967 10.1038/nature07976 PMC2676208

[11] Rumpelt HJ, Weisbach T (1978) Effect of cycloheximide on glucagon-induced autophagy. Quantitative examinations on hepatocytes in the rat. Am J Pathol 91: 49–56. 645821 PMC2018176

[12] Rez G, Kovacs J (1973) Prevention by cycloheximide of neutral red-induced formation of autophagic vacuoles and krinom granules in mouse pancreatic acinar cells. Virchows Arch B Cell Pathol 12: 123–132. 4197524 10.1007/BF02893992

[13] Beugnet A, Tee AR, Taylor PM, Proud CG (2003) Regulation of targets of mTOR (mammalian target of rapamycin) signalling by intracellular amino acid availability. Biochem J 372: 555–566. 12611592 10.1042/BJ20021266 PMC1223408

[14] Hara K, Yonezawa K, Weng QP, Kozlowski MT, Belham C, et al (1998) Amino acid sufficiency and mTOR regulate p70 S6 kinase and eIF-4E BP1 through a common effector mechanism. J Biol Chem 273: 14484–14494. 9603962 10.1074/jbc.273.23.14484

[15] Proud CG (2004) mTOR-mediated regulation of translation factors by amino acids. Biochem Biophys Res Commun 313: 429–436. 14684180 10.1016/j.bbrc.2003.07.015

[16] Kuma A, Hatano M, Matsui M, Yamamoto A, Nakaya H, et al (2004) The role of autophagy during the early neonatal starvation period. Nature 432: 1032–1036. 15525940 10.1038/nature03029

[17] Brasaemle DL, Barber T, Kimmel AR, Londos C (1997) Post-translational regulation of perilipin expression. Stabilization by stored intracellular neutral lipids. J Biol Chem 272: 9378–9387. 9083075 10.1074/jbc.272.14.9378

[18] Han J, Hajjar DP, Tauras JM, Nicholson AC (1999) Cellular cholesterol regulates expression of the macrophage type B scavenger receptor, CD36. J Lipid Res 40: 830–838. 10224152

[19] Hara A, Radin NS (1978) Lipid extraction of tissues with a low-toxicity solvent. Anal Biochem 90: 420–426. 727482 10.1016/0003-2697(78)90046-5

[20] Fewster ME, Burns BJ, Mead JF (1969) Quantitative densitometric thin-layer chromatography of lipids using copper acetate reagent. J Chromatogr 43: 120–126. 5802175 10.1016/s0021-9673(00)99173-8

[21] Abe-Dohmae S, Suzuki S, Wada Y, Aburatani H, Vance DE, et al (2000) Characterization of apolipoprotein-mediated HDL generation induced by cAMP in a murine macrophage cell line. Biochemistry 39: 11092–11099. 10998247 10.1021/bi0008175

[22] Cheng J, Fujita A, Ohsaki Y, Suzuki M, Shinohara Y, et al (2009) Quantitative electron microscopy shows uniform incorporation of triglycerides into existing lipid droplets. Histochem Cell Biol 132: 281–291. 19557427 10.1007/s00418-009-0615-z

[23] Pestka S (1971) Inhibitors of ribosome functions. Annu Rev Microbiol 25: 487–562. 4949424 10.1146/annurev.mi.25.100171.002415

[24] Czabany T, Wagner A, Zweytick D, Lohner K, Leitner E, et al (2008) Structural and biochemical properties of lipid particles from the yeast Saccharomyces cerevisiae. J Biol Chem 283: 17065–17074. 18430725 10.1074/jbc.M800401200

[25] McGookey DJ, Anderson RG (1983) Morphological characterization of the cholesteryl ester cycle in cultured mouse macrophage foam cells. J Cell Biol 97: 1156–1168. 6684660 10.1083/jcb.97.4.1156 PMC2112599

[26] Ohsaki Y, Maeda T, Maeda M, Tauchi-Sato K, Fujimoto T (2006) Recruitment of TIP47 to lipid droplets is controlled by the putative hydrophobic cleft. Biochem Biophys Res Commun 347: 279–287. 16808905 10.1016/j.bbrc.2006.06.074

[27] Duvel K, Yecies JL, Menon S, Raman P, Lipovsky AI, et al (2010) Activation of a metabolic gene regulatory network downstream of mTOR complex 1. Mol Cell 39: 171–183. 20670887 10.1016/j.molcel.2010.06.022 PMC2946786

[28] Jones KT, Greer ER, Pearce D, Ashrafi K (2009) Rictor/TORC2 regulates Caenorhabditis elegans fat storage, body size, and development through sgk-1. PLoS Biol 7: e60. 19260765 10.1371/journal.pbio.1000060 PMC2650726

[29] Soukas AA, Kane EA, Carr CE, Melo JA, Ruvkun G (2009) Rictor/TORC2 regulates fat metabolism, feeding, growth, and life span in Caenorhabditis elegans. Genes Dev 23: 496–511. 19240135 10.1101/gad.1775409 PMC2648650

[30] Escary JL, Choy HA, Reue K, Schotz MC (1998) Hormone-sensitive lipase overexpression increases cholesteryl ester hydrolysis in macrophage foam cells. Arterioscler Thromb Vasc Biol 18: 991–998. 9633942 10.1161/01.atv.18.6.991

[31] Ouimet M, Franklin V, Mak E, Liao X, Tabas I, et al (2011) Autophagy regulates cholesterol efflux from macrophage foam cells via lysosomal acid lipase. Cell Metab 13: 655–667. 21641547 10.1016/j.cmet.2011.03.023 PMC3257518

[32] Aslanidis C, Ries S, Fehringer P, Buchler C, Klima H, et al (1996) Genetic and biochemical evidence that CESD and Wolman disease are distinguished by residual lysosomal acid lipase activity. Genomics 33: 85–93. 8617513 10.1006/geno.1996.0162

[33] Carroll KS, Hanna J, Simon I, Krise J, Barbero P, et al (2001) Role of Rab9 GTPase in facilitating receptor recruitment by TIP47. Science 292: 1373–1376. 11359012 10.1126/science.1056791

[34] Bulankina AV, Deggerich A, Wenzel D, Mutenda K, Wittmann JG, et al (2009) TIP47 functions in the biogenesis of lipid droplets. J Cell Biol 185: 641–655. 19451273 10.1083/jcb.200812042 PMC2711566

[35] Bernales S, Papa FR, Walter P (2006) Intracellular signaling by the unfolded protein response. Annu Rev Cell Dev Biol 22: 487–508. 16822172 10.1146/annurev.cellbio.21.122303.120200

[36] Fei W, Wang H, Fu X, Bielby C, Yang H (2009) Conditions of endoplasmic reticulum stress stimulate lipid droplet formation in Saccharomyces cerevisiae. Biochem J 424: 61–67. 19708857 10.1042/BJ20090785

[37] Rutkowski DT, Wu J, Back SH, Callaghan MU, Ferris SP, et al (2008) UPR pathways combine to prevent hepatic steatosis caused by ER stress-mediated suppression of transcriptional master regulators. Dev Cell 15: 829–840. 19081072 10.1016/j.devcel.2008.10.015 PMC2923556

