# Supplementary figures and images for: Translation Inhibitors Induce Formation of Cholesterol Ester-Rich Lipid Droplets
Source: PLoS One. 2012 Aug 3;7(8):e42379. doi: 10.1371/journal.pone.0042379 (PMC3411751; doi:10.1371/journal.pone.0042379)

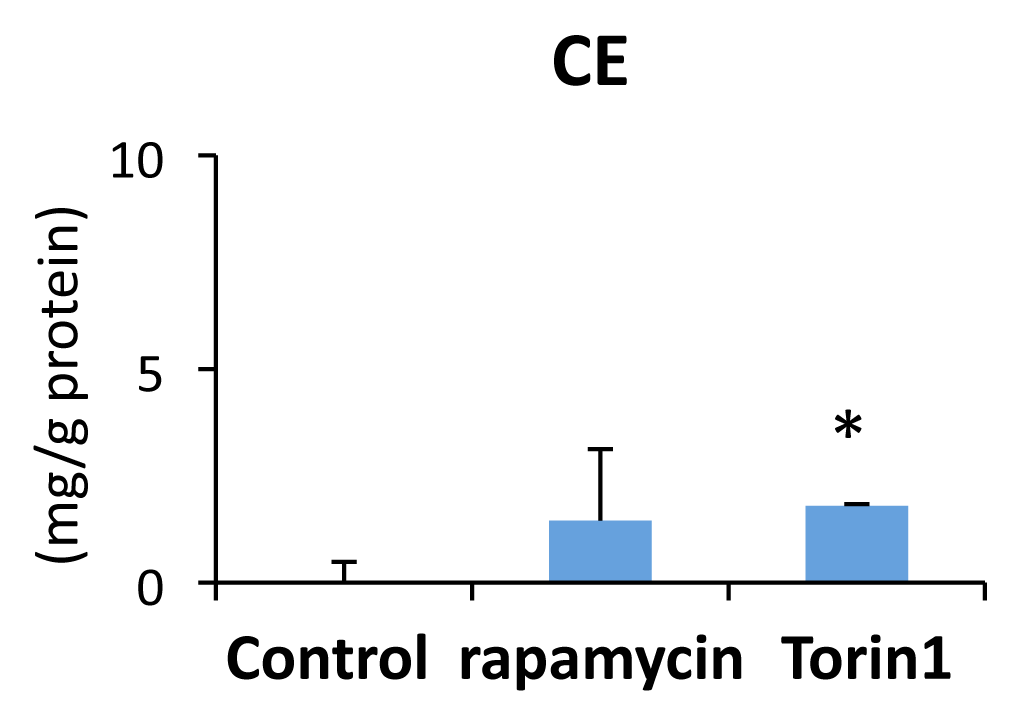

Supplement: Figure S1 — Quantification of CE. 3Y1 cells were treated with or without 0.4 µM rapamycin or 0.25 µM Torin1 for 18 hr. CE showed an increase caused by either rapamycin or Torin1, but only the increase caused by Torin1 was significant (*p<0.05; Student’s t test). Mean ± SD is shown. (TIF) [file pone.0042379.s001.tif]
